# Harm Reduction Contingency Management for Stimulant Use Reduction and Antiretroviral Therapy Adherence in HIV Primary Care: Protocol for an Implementation Effectiveness Study

**DOI:** 10.2196/67292

**Published:** 2025-08-18

**Authors:** Gabriela Steiner, Stefan Baral, Elise D Riley, Steven Shoptaw, Gabriel Chamie, Kate Roberts, Lauren Suchman, Kelly Knight, Monica Gandhi, Phillip Coffin, Ayesha Appa

**Affiliations:** 1 University of California, San Francisco San Francisco, CA United States; 2 Johns Hopkins University Baltimore, MD United States; 3 University of California, Los Angeles Los Angeles, CA United States; 4 Bryn Mawr Graduate School of Social Work and Social Research Philadelphia, PA United States; 5 San Francisco Department of Public Health San Francisco, CA United States

**Keywords:** substance use disorder, motivation, drug users, central nervous system stimulants, medication adherence

## Abstract

**Background:**

Stimulant use disorder has been linked with medication nonadherence and mortality among people living with HIV. Contingency management (CM) is a strategy incentivizing measurable behavior change that is recommended as the first-line treatment for stimulant use disorder and can support antiretroviral therapy (ART) adherence. However, CM is not widely implemented, in part due to feasibility concerns. Although reductions in substance use short of full abstinence can improve health outcomes, CM programs typically target complete abstinence from stimulant use rather than reduction. To optimize care for safety-net populations living with comorbid stimulant use disorder and HIV, we designed a novel CM program incentivizing both stimulant use reduction and ART adherence in the HIV ambulatory setting.

**Objective:**

We aimed to (1) evaluate the feasibility of once-weekly CM in safety-net HIV ambulatory care and (2) assess the acceptability of CM among participants and care providers.

**Methods:**

We will conduct a pilot, single-arm, hybrid implementation effectiveness trial offering a novel CM intervention in 2 low-barrier, ambulatory HIV clinics. Patients with stimulant use disorder and suboptimal ART adherence will be offered 12 weeks of once-weekly CM including incentives for positive-tenofovir and negative-stimulant results on urine point-of-care assays. We will assess stimulant use once weekly using tests with a 4-day detection window, allowing participants to use stimulants during select days of the week but earn incentives by reducing their frequency of use from near-daily use. We will assess feasibility and acceptability using quantitative process methods and qualitative in-depth interviews, guided by the RE-AIM (reach, effectiveness, acceptability, implementation, and maintenance) evaluation framework. We will define preliminary effectiveness by proportion of stimulant-negative and tenofovir-positive urine tests, as well as changes in HIV viral suppression before and after participation.

**Results:**

Recruitment and CM visits have concluded as of September 2024. Quantitative and qualitative evaluation is underway and is expected to continue through October 2025.

**Conclusions:**

This novel CM program offers dual incentives targeting stimulant use reduction and ART adherence. We hypothesize that incentivizing stimulant use reduction is acceptable to our target safety-net population. Demonstration of a feasible, acceptable model may serve as a first step toward wider use of stimulant-reduction CM in the safety-net setting.

**Trial Registration:**

ClinicalTrials.gov NCT06564792; https://clinicaltrials.gov/study/NCT06564792

**International Registered Report Identifier (IRRID):**

DERR1-10.2196/67292

## Introduction

### Background

Stimulant use disorder continues to rise as a devastating public health issue in the United States, accounting for more than 57,000 deaths in 2022—a near 5-fold increase since 2015 [1] Among people living with HIV, stimulant use is particularly prevalent (>5% to 15% in the general population of people with and 30% in safety-net patient populations living with HIV) and represents a key challenge for maintaining overall health [2-4]. Both methamphetamine and cocaine have been repeatedly associated with antiretroviral therapy (ART) nonadherence, HIV viremia, and progression to AIDS; by some estimates, people living with HIV using stimulants demonstrate double the odds of ART nonadherence compared to those not using stimulants [5-9].

Contingency management (CM) is the first-line treatment for stimulant (methamphetamine and cocaine) use disorder and has also been used to promote ART adherence for people living with HIV [10-15]. This behavioral treatment—in which patients receive tangible incentives for demonstrating abstinence (eg, measured by drug-negative urine)—is highly efficacious, supported by a multitude of randomized controlled trials [10,16,17]. In addition, research suggests that CM can also be efficacious for ART adherence, although these studies were limited by the potential inaccuracy of indirect adherence measurements (eg, electronic pill bottle cap monitoring does not equate to ingested medication) [11-15].

Despite its demonstrated efficacy, the uptake of CM remains limited in both safety-net and ambulatory settings. While these settings represent the main source of medical care for many high-need patients, feasibility concerns have largely impeded CM implementation. Frequently described barriers include insufficient training and awareness, logistic concerns related to program execution, cost and resource intensiveness of programs requiring multiple weekly visits, and staff reluctance to provide financial incentives [18-21]. In addition, addressing syndemic behaviors simultaneously (by incentivizing both stimulant use reduction and ART adherence through a single “multitarget” intervention) is an important opportunity to seize. To effectively reach people living with HIV who are most in need of this treatment, there is an urgent need for feasible CM models that target stimulant use and ART adherence in ambulatory safety-net settings.

Leveraging harm reduction principles will be essential for addressing the needs of safety-net populations with comorbid HIV and stimulant use disorder. Abstinence from stimulants can be an unrealistic, unwanted, or a less proximate goal for people with severe stimulant use disorder, who may be using daily or chronically [22]. Efforts to emphasize stimulant use reduction rather than abstinence have shown promise in promoting overall health; for example, in a secondary analysis of 13 randomized controlled trials, reduced use of methamphetamine and cocaine was associated with global improvement of psychosocial functioning and severity of drug-related problems, as well as improved depression and fewer drug-seeking behaviors [23]. Although CM is traditionally structured to promote abstinence, new models that incentivize stimulant use reduction and other positive health behaviors may be more aligned with patient goals and needs in the safety-net setting.

To optimize care for safety-net populations living with comorbid stimulant use disorder and HIV, we designed a novel CM program incentivizing both stimulant use reduction and ART adherence in the HIV ambulatory setting, called CoMBo (Contingency Management for Both HIV and Stimulant Use Disorder). CoMBo involves point-of-care (POC) tests that detect stimulant use within a 3- to 4-day window and offer once-weekly visits in contrast to traditional CM programs that test urine 2 to 3 times per week to confirm abstinence. In addition, we use a novel POC, a urine-based tenofovir assay that allows for real-time assessment of adherence to tenofovir-based ART [24,25]. This technology enables simultaneous assessment of tenofovir adherence and stimulant use by using one urine sample, facilitating a truly paired intervention.

In this paper, we present a full description of an implementation effectiveness pilot delivering CM with paired incentives in the HIV ambulatory setting and describe a protocol for the quantitative and qualitative evaluation of program feasibility and acceptability (the CoMBo study). We hypothesize that (1) this program will be acceptable to staff and participants and (2) offering once-weekly CM (as opposed to multiple-times per week) to achieve stimulant reduction (as opposed to abstinence) in low-barrier medical homes of people living with HIV will be feasible.

### CoMBo Study Objectives

The following are the objectives of this study:

1. To evaluate the feasibility of once-weekly CM in safety-net HIV ambulatory care to decrease stimulant use and improve ART adherence
2. To qualitatively assess the acceptability of CM offered in safety-net HIV ambulatory care to participants and care providers

## Methods

### CoMBo Study Design

We will conduct a pilot, single-arm, hybrid implementation effectiveness trial of dual-incentive CM targeting stimulant use reduction and improved ART adherence in 2 low-barrier, ambulatory HIV clinics. We use the Behavior Change Wheel framework to tailor this intervention to meet the needs of our target safety-net population (Table 1). This study design will allow for the investigation of implementation feasibility in our target setting, while also gathering data on the intervention’s impact on target behaviors. The study will occur over a period of 9 months; patient recruitment will occur during the first 6 months of the study on a rolling basis. Following this pilot, we will solicit feedback on intervention acceptability using in-depth qualitative interviews with participants and clinic staff. The study has been registered as a clinical trial (ClinicalTrials.gov NCT06564792).

**Table 1:** Formative intervention components for targeting behavior change in safety-net ambulatory care, as described by the Behavior Change Wheel framework [26].

Source of behavior	Example of barriers to behavior change	Intervention component	How will intervention component facilitate behavior change?
Motivation (reflective and automatic)	Complete abstinence following a long period of dependence may be unrealistic, intimidating, or unwanted	1. Assess stimulant use reduction using once weekly using point-of-care tests with a 2- to 4-day detection window	Incentivization: create expectation of reward
Motivation (reflective)	Internalized stigma around psychosocial factors (eg, substance use and housing instability) leads to low self-belief in ability to change behavior	2. Engage in motivational interviewing, emphasizing self-talk and goal setting and providing social support	Persuasion: induce positive or negative feelings to stimulate actions
Opportunity (physical and social)	Limited resources to schedule and keep consistent in-person appointments (eg, limited access to phone, transportation)	3. Offer drop-in, one-on-one visits	Environmental restructuring: change physical and social context

### Study Setting

This study will be conducted at 2 subclinics at San Francisco General Hospital’s Ward 86, one of the oldest HIV clinics in the country serving more than 2600 people living with HIV in the county safety net. Ward 86 provides wraparound care with co-located behavioral health, social work, case management, laboratory, and pharmacy services. The Women’s Clinic is an interdisciplinary, team-based model of care providing HIV, substance use disorder, drop-in services, and mental health care to people identifying as cis- or transwomen living with HIV at Ward 86, serving 234 patients as of July 2022. The POP-UP clinic is another interdisciplinary, team-based clinical program within Ward 86 providing low-barrier, drop-in primary care to patients with HIV experiencing homelessness. At least 30% of the Ward 86 population report regular stimulant use, with approximately 40% of Women’s Clinic patients and 90% of POP-UP patients reporting stimulant use. By co-locating CM into low-barrier medical homes for vulnerable people living with HIV, we aim to decrease barriers to CM engagement for patients with comorbid stimulant use disorder and HIV.

### Study Population and Inclusion and Exclusion Criteria

Recruitment, enrollment, and study procedures will occur at Ward 86, with targeted recruitment from the aforementioned clinics. We aim to recruit participants living with HIV who (1) are on tenofovir-based ART regimens, (2) are diagnosed with severe stimulant use disorder (according to the *Diagnostic and Statistical Manual of Mental Disorders, Fifth Edition* criteria, measured with the Structured Clinical Interview for DSM-5, using at least near daily), (3) have suboptimal ART adherence (defined as at least one instance of HIV viral load over 200 copies/mL in the preceding 18 months or reporting periods of discontinuation within last 18 months), (4) are interested in reducing frequency of stimulant use and improving ART adherence, and (5) speak either English or Spanish [26]. On the basis of the estimated prevalence of comorbid stimulant use disorder and HIV within our target safety net, we aim to recruit approximately 40 participants.

### Recruitment and Informed Consent

Primary care providers within the safety net may refer participants via email or direct messaging through the electronic health record system (Epic), and participants may also spontaneously walk-in for enrollment without a referral. Eligibility criteria are assessed via phone (for referrals) or in-person (for walk-ins) and confirmed via the electronic health record (last detectable viral load, confirmation of stimulant use disorder diagnosis, and ART regimen) before enrollment. Participants who meet the eligibility criteria are offered an enrollment visit, in which they provide informed consent and complete baseline assessments.

### Intervention

#### Description

**Figure 1:**
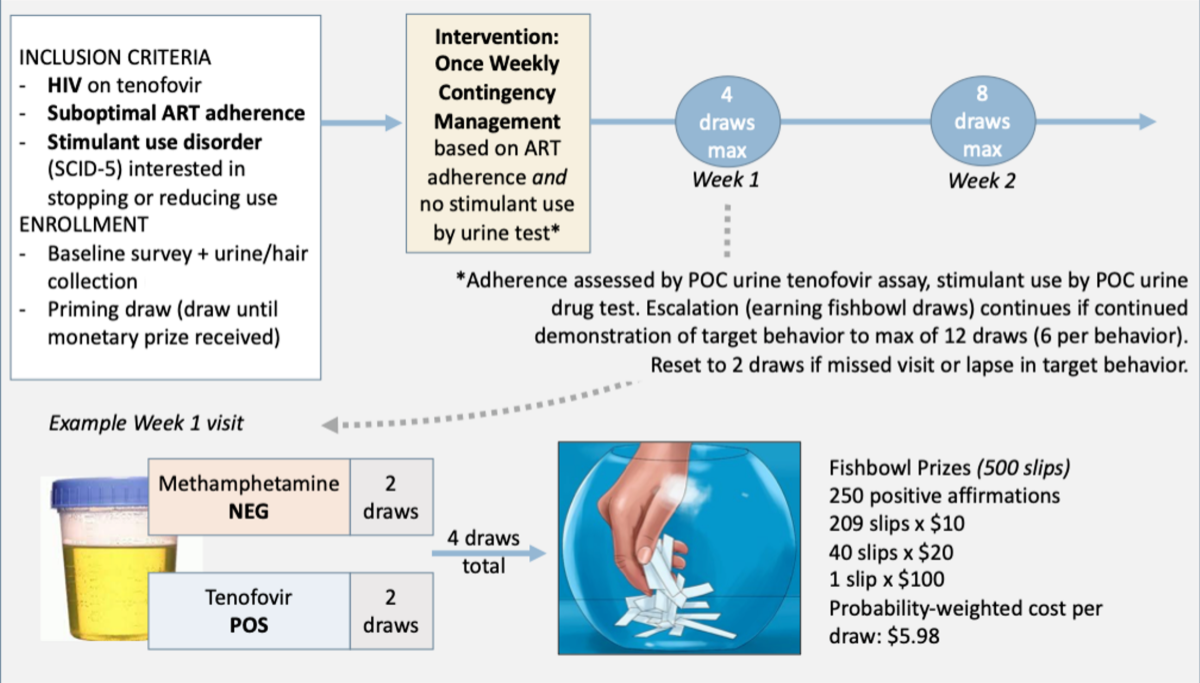
Intervention description. ART: antiretroviral therapy; NEG: negative; POC: point of care; POS: positive; SCID-5: Structured Clinical Interview for DSM-5.

Enrolled participants who have provided informed consent will complete baseline surveys and urine testing (Table 2), then come to the clinic (in-person) once weekly for 12 weeks. During CM visits, participants will engage in motivational interviewing (MI) and provide urine for POC tenofovir and stimulant testing, with escalating incentives for a longer duration of stimulant-free and tenofovir-containing urine (Figure 1) [27]. Of note, incentives will not require both behavioral targets to be met. For example, if a participant’s urine tests positive for tenofovir but also for cocaine, they would receive draws for tenofovir but not for stimulants. At the next study visit, the stimulant-related draws would reset to 2 (if no stimulant in urine) with a rapid reset procedure for each target behavior following a lapse in target behavior (or missing sample).

**Table 2:** Assessment variables and schedule for dual-incentive contingency management in HIV primary care.

Domain and assessment			Stage of collection		
			Baseline		Study visits
**Sociodemographics**					
	Gender, age, housing status, and race or ethnicity	✓			
**Substance use history**					
	Substances currently using	✓			
	Route of use	✓			
	Overdose history, from what substances?				
	7-d timeline follow-back^a^	✓		✓	
	Severity of dependence scale^a^	✓			
**HIV history**					
	Last detectable viral load	✓		✓	
	3-point Medication Adherence Assessment^a^	✓			
	“Did you take [medication] more, less, or the same this week compared to last week?”			✓	
**Point-of-care results**					
	Tenofovir detected	✓		✓	
	Stimulants detected	✓		✓	
	Other substances detected	✓		✓	
**Visit characteristics**					
	Length of visit	✓		✓	
	Number of incentives earned			✓	
	Amount earned	✓		✓	

^a^Use of the validated assessment tool.

All visits will occur in-person in clinic on the same day of the week. Participants will be invited to drop-in for study appointments anytime during clinic hours (9 AM to 5 PM). Visits are estimated to last approximately 20 minutes.

The following key tenets of CM will be adhered to: (1) frequently monitoring the behavior of interest (eg, stimulant use and adherence assessed weekly); (2) tangible, immediate positive reinforcement after confirmation of behavior of interest (eg, POC testing allows for immediate provision of incentives if indicated); and (3) positive reinforcement (ie, incentives) withheld if behavior of interest is not demonstrated [21]. We will use the prize method for reinforcement provision (also known as the “fishbowl method,” established by CM authority Dr Nancy Petry), in which participants earn an escalating number of draws from a fishbowl containing paper slips with variable monetary value [28,29]. As specified by distribution of paper slips, patients have a 50% chance of drawing a monetary incentive and 50% chance of drawing an affirmation with an encouraging message (no monetary value; Figure 1). In keeping with this method, participants will receive a “priming draw” at their enrollment visit, in which they draw from the fishbowl until they receive a monetary prize, regardless of their POC results. If a participant misses a visit or does not provide evidence for the target behaviors, the number of draws related to that behavior resets to zero (ie, stimulant positive urine will reset stimulant-related draws to zero).

#### Theory for Formative Components

To customize the CM intervention to our target safety-net population, we used the Behavior Change Wheel framework to identify population-specific barriers to behavior change and created intervention components to address these barriers (Table 1). These formative components are discussed in the subsequent sections.

#### Incentivizing Stimulant Use Reduction

We hypothesize that incentivizing stimulant use reduction rather than abstinence will be acceptable to our target safety-net population with comorbid stimulant use disorder and HIV. One challenge to incentivizing stimulant use reduction is that no POC tests measure quantitative levels of cocaine or methamphetamine and thus cannot determine if one positive result represents a reduction or increase in use compared to another positive result. To overcome this challenge, we will assess stimulant use once weekly using POC tests with an approximate 4-day detection window. This would theoretically allow participants to continue using substances some days of the week, but still test negative for stimulants on POC tests if they do not use in the days preceding testing. Given that (per inclusion criteria), participants endorse using stimulants most days of the week (if not daily), we theorize that a negative result will constitute a true sign of use reduction.

#### Offering Drop-In, Individual Visits

The need to adhere with scheduled clinic appointments is a frequently cited barrier to engagement in care for vulnerable populations, including people living with HIV. Emerging models of care have shown that offering low-threshold, drop-in visits without an appointment can circumvent this barrier to achieve robust improvement in care engagement and target health outcomes; one such model is demonstrated in the POP-UP clinic, one of this study’s 2 host clinics, wherein more than 50% of enrolled, viremic patients experiencing homelessness achieved viral suppression within 6 months [30,31]. In keeping with low-barrier principles, we expect that offering drop-in visits will optimize engagement for safety-net patients with complex psychosocial factors.

#### Incorporating MI

MI is a psychosocial interviewing technique that aims to enhance intrinsic motivation by eliciting statements that amplify and reinforce a patient’s commitment for behavioral change [32]. Several meta-analyses have shown that interventions using MI have had some effect on reducing substance use (including stimulants) and that pairing MI with CM interventions may lead to more durable behavior change [33]. In addition, integrating MI into CM study visits can help promote relationship building between patients and CM providers, which we expect will be particularly important for cultivating trust among patients with negative past health care experiences related to substance use or HIV-related stigma [33].

### CM Provider and Training

One CM provider will be trained to lead all CM activities with supervision by the principal investigator, an addiction medicine specialist. Training includes (1) POC device training; (2) completing the free, virtual “Contingency Management Overview Training” offered by the Recovery Incentives Program at the University of California, Los Angeles; (3) addiction medicine–specific intensive MI through a 2-day training workshop [34].

### Materials

At enrollment, participants will receive a visual schema describing the incentive structure and indicating where or when they may come in for CM visits (Multimedia Appendix 1).

**Figure 2:**
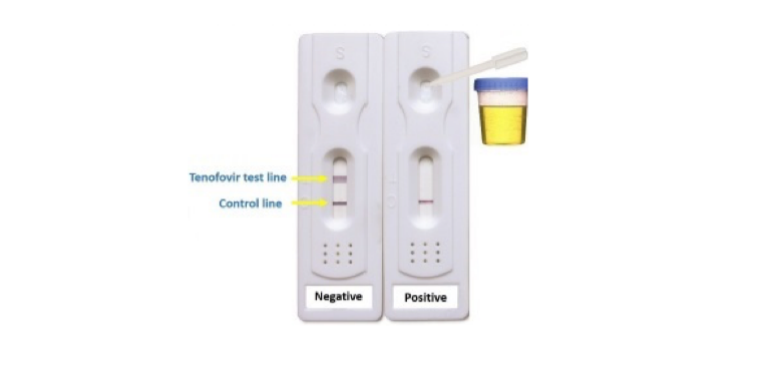
Novel point-of-care urine tenofovir test.

The materials required for conducting study visits include fishbowl with paper slip incentives (Figure 1), stimulant POC tests, tenofovir POC tests (Figure 2), gift cards, and sign-out sheets (to confirm receipt of gift cards and record barcodes, as required by our funding mechanism), and timeline follow-back form (Multimedia Appendix 1).

The number of incentives earned during each visit will be automatically calculated via REDCap (Research Electronic Data Capture; Vanderbilt University; calculated field using special functions) after input of POC results.

### Assessment and Data Collection

All data will be captured and stored using REDCap electronic data capture tools hosted at University of California, San Francisco [35,36]. Most data metrics (described in Table 2) will be extracted from patient interviews during baseline and study visits, although viral loads will be extracted from patient’s electronic medical record.

Baseline assessments (Table 2) will extract patient variables relating to sociodemographics, substance use history, and HIV history, including HIV viral load before participation. We will also collect POC results at baseline (although no incentives will be earned from these results), perform 7-day timeline follow-back to assess baseline frequency of stimulant use, and record visit-related characteristics (length of visit and amount in US $ received through a priming draw; Multimedia Appendix 1) [37].

Visit assessments will assess recent stimulant use and ART adherence, capture POC results, and record visit characteristics (including number of incentives earned). At each visit, we will objectively assess each target behavior through POC testing (used to calculate incentives), subjectively assess stimulant use using 7-day timeline follow-back, and subjectively assess ART adherence using a 3-point Likert scale question (Table 2).

Upon intervention conclusion after 12 study visits, we will record the viral load most proximate to the patient’s last attended visit.

### Evaluation Frameworks and Outcomes

#### Quantitative Evaluation

We will leverage the RE-AIM (reach, effectiveness, adoption, implementation, and maintenance) framework (Table 3) to quantitatively evaluate feasibility, reach, effectiveness, and intervention implementation.

Per our first study objective, we aim to evaluate the feasibility of implementing this intervention in the safety-net, ambulatory HIV care setting, which we will assess using various domains of the RE-AIM evaluation framework. Our primary outcome will be defined via engagement: 50% CM visit attendance will indicate a threshold for program feasibility. Secondary feasibility outcomes will include number of visits performed, average visit time, and monetary value earned per participant, which map to domains (Table 3). Metrics related to “reach” will assess if the proposed implementation model will be used by a diverse patient population with complex sociomedical needs (as intended), reflecting both the utility and accessibility of this program. “Effectiveness” metrics will seek to establish if the intervention modified the target behavior changes, leading to reduced stimulant use, improved ART adherence, and undetectable HIV viral loads. “Implementation” metrics will describe the costs required to start and maintain this program during the study period (8 months). Our secondary outcome is preliminary effectiveness, which we will determine using proportion of stimulant-negative and tenofovir-positive urine tests, as well as changes in HIV viral suppression before and after participation. We will report missing data and impute missing viral load data as >200 copies/mL.

**Table 3:** RE-AIM (reach, effectiveness, adoption, implementation, and maintenance) framework evaluation of dual-incentive contingency management targeting antiretroviral therapy adherence and stimulant use in ambulatory HIV care.

Dimension	Question	Outcomes
Reach	Will safety-net patients living with HIV and stimulant use disorder enroll in this program? Will they return after enrollment?	Sociodemographics Substance use and HIV histories Proportion of study visits attended
Effectiveness	Will participants reduce stimulant use? Will participants improve tenofovir adherence?	Weekly point-of-care results and 7-d timeline follow-back
Adoption	Will offering this program in the ambulatory HIV care setting be acceptable to staff and participants? What changes, if any, would make the program more or less acceptable?	Qualitative evaluation
Implementation	What were the monetary and other resource costs of program implementation?	Length of visits Total gift cards provided (amount in US $) Proportion of incentives earned for stimulant use versus tenofovir adherence Number of point-of-care tests used Time spent on participant outreach
Maintenance	Not assessed in this pilot	—^a^

^a^Not applicable.

#### Qualitative Evaluation

Per our second study objective, we will qualitatively evaluate program acceptability to participants and health care staff following the Consolidated Framework for Implementation Research (CFIR). Constructs describing acceptability are encompassed by “adoption” and seek to describe circumstances and factors that influence intervention engagement among participants and synergy within the ambulatory HIV care ecosystem.

### Sampling and Recruitment

Participants will be asked to engage in the qualitative evaluation following study completion (week 12 from enrollment), during either the end of their final study visit or when they return to the home clinic for routine care. Any participant who enrolled and completed at least one study visit will be eligible to enroll. Eligible staff will be identified by the study coordinator, who will recommend staff who meaningfully contributed feedback regarding the pilot rollout or who interfaced with the pilot’s day-to-day functioning. For example, we anticipate the clinic’s head nurses and primary social workers to meet this criteria.

### Data Collection

Interviews will occur at each home clinic during clinic hours. They will be conducted by 1 to 2 trained researchers with extensive experience conducting qualitative interviews and background in HIV or addiction medicine. Participants will be consented by the interviewer before the interview. In-depth interview guides have been developed using the CFIR framework in order to systematically evaluate setting, intervention, individual, and process characteristics influencing program acceptability (Table 4; Multimedia Appendices 2 and 3). All interviews will be recorded; the interviewer will refrain from using identifying language during the record process, for example, by using participants’ study ID numbers instead of their name. Interviews are estimated to last approximately 45 minutes. Each interviewee will receive US $50 worth of gift cards for their participation.

**Table 4:** Consolidated Framework for Implementation Research (CFIR) framework for qualitative evaluation of program acceptability in low-barrier, ambulatory HIV care.

Target interviewees, CFIR domain, and CFIR construct				Question examples
**Care providers and staff who were involved with study participants (n=10)**				
	**Inner setting**			
		Compatibility	How compatible was this intervention with existing work processes at the clinic?	
		Tension for change	Is there a need for this intervention?	
		Relative priority	How important is it to provide this intervention, compared with other priorities?	
		Networks and communication	How have you referred participants?	
	**Characteristics of the individual**			
		Knowledge and beliefs	How do you feel about this intervention being offered at [clinic name]?	
**Participants who attended >50% of study visits (N=7); participants who attended <50% of study visits (N=7)**				
	**Intervention characteristics**			
		Relative advantage	How would your experience be different if you had to schedule visits instead of dropping-in?	
	**Process**			
		Engaging	How easy or difficult was it to participate in the program?	
		Executing	What did you think about getting urine results for both stimulants and tenofovir?	
		Reflecting and evaluation	How do you think you benefited from this program?	

### Participant Interviews

We will create a purposive sample of 14 trial participants, with priority recruitment of at least 7 participants who completed fewer than 50% of CM visits and 7 participants who completed more than 50% of CM visits (Table 4). This sample size will largely depend on the number of participants recruited, but we aim to interview approximately 30% of our total target sample size (ie, 14 participants in a pool of 40). Interviews will seek to (1) understand participant experiences in the primary care–based, stimulant, and adherence-targeted CM intervention and (2) explore how multilevel factors might positively and negatively influence engagement with multitarget CM (eg, role of relationships in the clinic, and concurrent synergistic clinical goals such as gender-affirming surgery, intervention factors such as drop-in visits, and reinforcement schedule, etc). We are secondarily interested in identifying how participant goals for stimulant use reduction are related to HIV management, if at all, and how drivers of stimulant use and additional determinants of health (eg, homelessness, intimate relationships, etc) interact with HIV and stimulant use disorder management.

### Care Provider and Staff Interviews

We will also conduct qualitative interviews with 5 to 10 care providers and staff who were involved in participants’ care or who interfaced with the intervention’s activities (eg, referred participants and assisted with program logistics). This final sample size will depend on how many care providers meaningfully interact with this pilot; we have identified a minimum of 5 care providers whose roles interface with the intervention, and we expect this number to increase as we integrate the problem into the everyday functioning of our target clinics. We seek to investigate perceived appropriateness of dual-incentive CM in the low-barrier HIV care context, elucidating perspectives on (1) the intervention’s compatibility with existing clinic processes, (2) how well the intervention met clinic needs and relative importance of these needs, and (3) changes to promote long-term sustainability.

### Analysis Plan

#### Quantitative Evaluation

We will analyze reach and implementation metrics (Table 3) using basic descriptive statistics. For effectiveness, we will use descriptive statistics to describe POC results, including the proportion of negative-stimulant and positive-tenofovir tests each week and cumulatively overall and the number of participants that test negative for stimulants or positive for tenofovir at least once. We will also report the average frequency of use as captured by weekly timeline follow-back and compare average use at baseline versus study visit 12 (ie, the final visit) using a paired 2-tailed *t* test. We will use the McNemar test to compare the proportion of participants with HIV viral loads less than 200 copies/mL before and after CM participation.

#### Qualitative Evaluation

To analyze our qualitative data, we will follow the main principles of “scientifically descriptive” thematic analysis as defined by Finlay [38] and further elucidated by Braun and Clarke [39]. Audio recordings will be professionally transcribed into Microsoft Word documents and saved on a secure, password-protected, cloud-based folder that is only accessible to the research team. As traditional qualitative coding is a well-documented, lengthy process, we will use the framework analysis approach to efficiently generate findings with a small team [40,41]. We will first develop a spreadsheet in which we plot the key CFIR constructs outlined in Table 4 across the x-axis and individual research participants across the y-axis. One of the researchers who conducted both patient and care provider interviews (LS) will then review each transcript and copy and paste individual excerpts into the appropriate columns in the spreadsheet. For example, if a patient mentions that they would have trouble keeping an appointment if that was required for participation in the study, this information will be recorded in the “Relative Advantage” column. To keep with the definition of scientifically descriptive thematic analysis by Braun and Clarke [39] and ensure confirmability of findings, a second researcher on the team (AA) will then cross-check a select subset of transcripts and meet with LS to discuss any discrepancies and reach consensus. To enhance reflexivity, the analysts will maintain awareness of how their academic backgrounds may shape data collection and interpretation, ensuring that participants’ experiences with stimulant use disorder and HIV are centered in the analysis.

### Ethical Considerations

This study was approved by the Institutional Review Board at University of California, San Francisco (23-38747). Written informed consent will be obtained from participants before enrollment, consenting to study activities, review of electronic medical records by research staff, and secondary analyses using study data. Consents will be available in English and Spanish (Multimedia Appendices 4 and 5). Participants may opt out of the study at any point.

To maintain participant privacy, any data relating to the study will be stored in either REDCap or an encrypted Box folder hosted by the University of California, San Francisco. Only members of the research team will have access to any participant data stored in REDCap. Study data will not be recorded or linked to the patients’ electronic medical records. Audit of gift cards will be performed by the San Francisco Department of Public Health, per the Controller’s Office protocol. No participants currently incarcerated will be enrolled in this study.

Compensation will be provided for participation in the qualitative evaluation and will consist of a US $50 gift card that can be used at most grocery stores.

## Results

As of September 26, 2024, a total of 37 patients have been recruited and they completed 12 weeks of CM. Analysis of quantitative metrics for RE-AIM evaluation has concluded as of June 2025. Qualitative evaluation has begun, with 10 participants and 7 staff interviewed. Interviews concluded as of January 2025, and analysis is underway.

## Discussion

This work may demonstrate that once-weekly CM targeting both stimulant use disorder and ART adherence can be feasibly implemented in safety-net ambulatory care settings, as defined by the RE-AIM framework. In addition, we may demonstrate through qualitative analysis that such a program is acceptable to both patients and care providers who work in safety-net ambulatory care settings. We anticipate that this single-arm trial will inform any subsequent larger trials investigating the efficacy of offering CM in the HIV primary care setting.

Implementing CM interventions in real-world settings is a critical first step toward reducing morbidity and mortality from stimulant use disorder. Implementation in safety net, ambulatory HIV care—with targeted sampling among patients experiencing homelessness—will help meet the urgent needs of medically complex individuals at high risk for complications of substance use. This protocol offers the opportunity to address syndemic behaviors through one intervention—namely, the related behaviors of stimulant use and ART nonadherence. The success of this pilot would demonstrate the feasibility of offering this evidence-based treatment in HIV ambulatory care and provide a scalable model for implementation in similar safety-net settings. By assessing acceptability through qualitative interviews, we aim to center community voices and align this program with the goals, needs, and lived experiences of people living with HIV and stimulant use disorder.

While abstinence from stimulant use is thought to provide the most clinical benefit (albeit less feasible), use reduction is emerging as an important and meaningful treatment outcome for patients with stimulant use disorder [42]. Moreover, as the field of addiction is evolving to understand addiction as nuanced and multifaceted, we must offer a wider range of treatment implementation options that meet a diversity of patient goals, which may encompass both abstinence-based and reduction-based approaches. In the HIV space specifically, holistic care should address substance use and its related health behaviors, such as reduced rates of ART adherence. Emphasis on reducing stimulant use for people living with HIV and stimulant use disorder may serve as a vehicle to improve overall health among highly susceptible patients.

The use of tenofovir POC assays represents an important advancement, but the inability to rapidly test other forms of ART limits this program to patients on tenofovir-based regimens. This requirement excludes individuals on long-acting ART that are not tenofovir based, although other incentive structures to support injection adherence could be considered. In addition, no POC tests can rapidly quantify stimulant use, thus we rely on indirect methods for assessing stimulant use reduction. While this study targets the enrollment of patients with severe, frequent stimulant use, this program may not be optimally effective for individuals who desire abstinence or with less frequent stimulant use at baseline. Finally, we rely on participant self-report to establish ART nonadherence, which is subject to bias.

This study describes a novel CM program that (1) offers dual incentives targeting the syndemic behaviors of stimulant use and ART nonadherence, (2) uses a novel tenofovir urine assay for POC ART testing, and (3) incentivizes stimulant use reduction. In addition, we describe a protocol for evaluating implementation feasibility in HIV ambulatory care and acceptability to participants and staff. Demonstration of a feasible model that is acceptable to participants with HIV and stimulant use disorder may serve as a first step toward wider use of CM in the safety-net setting and can help meet critical needs in this population.

## Appendix

Multimedia Appendix 1 HIV and stimulant use contingency management study details.

Multimedia Appendix 2 Patient-facing interview guide.

Multimedia Appendix 3 Staff-facing interview guide.

Multimedia Appendix 4 Consent form in English.

Multimedia Appendix 5 Consent form in Spanish.

## Abbreviations

ART: antiretroviral therapy
CoMBo: Contingency Management for Both HIV and Stimulant Use Disorder
CFIR: Consolidated Framework for Implementation Research
CM: contingency management
MI: motivational interviewing
POC: point of care
REDCap: Research Electronic Data Capture
RE-AIM: reach, effectiveness, acceptability, implementation, and maintenance
