# Supplementary figures and images for: Harm Reduction Contingency Management for Stimulant Use Reduction and Antiretroviral Therapy Adherence in HIV Primary Care: Protocol for an Implementation Effectiveness Study
Source: JMIR Res Protoc. 2025 Aug 18;14:e67292. doi: 10.2196/67292 (PMC12402737; doi:10.2196/67292)

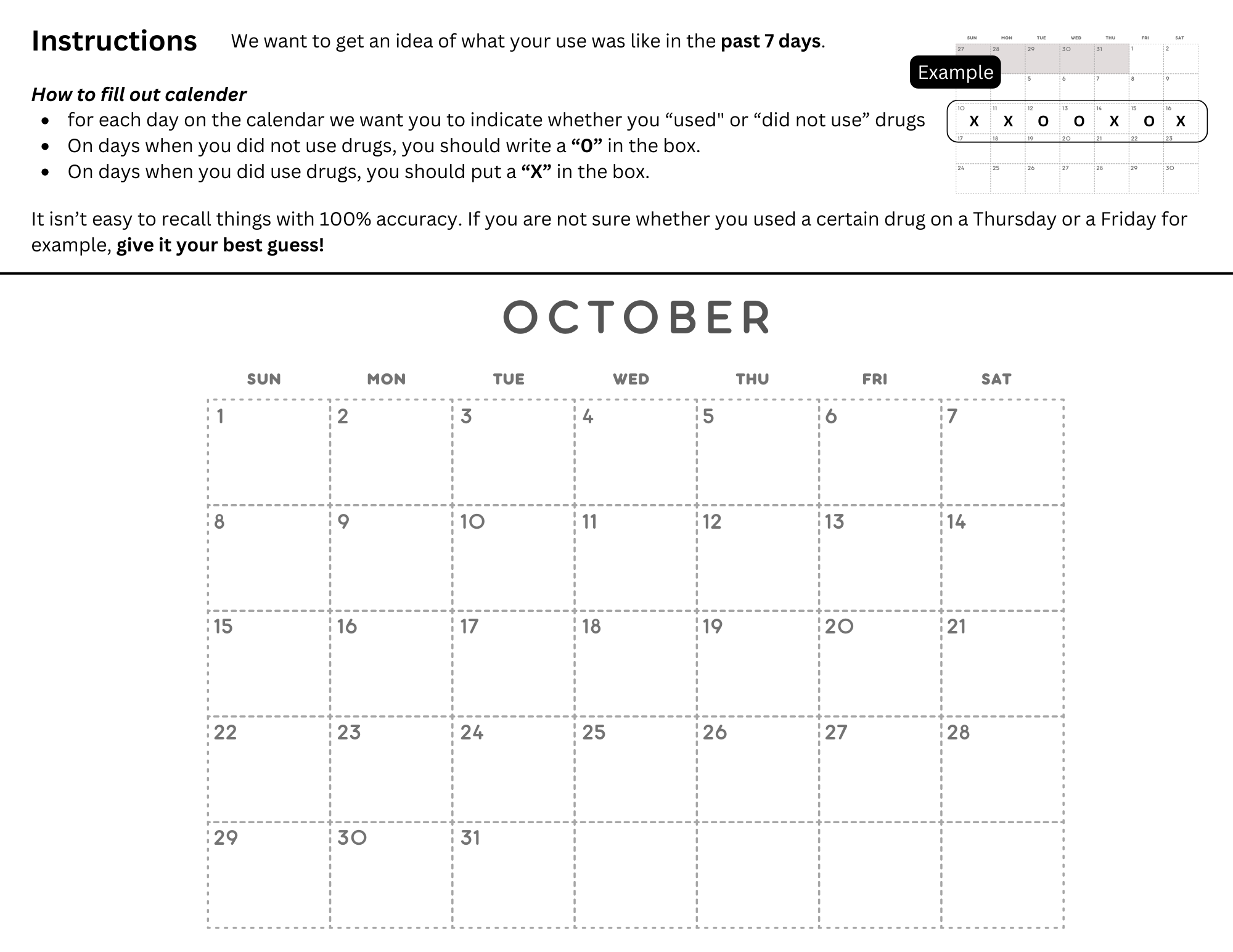

Supplement: Multimedia Appendix 1 [file resprot_v14i1e67292_app1.png]
