# Supplementary material for: Harm Reduction Contingency Management for Stimulant Use Reduction and Antiretroviral Therapy Adherence in HIV Primary Care: Protocol for an Implementation Effectiveness Study
Source: JMIR Res Protoc. 2025 Aug 18;14:e67292. doi: 10.2196/67292 (PMC12402737; doi:10.2196/67292)
Supplement: Multimedia Appendix 2 [file resprot_v14i1e67292_app2.docx]

## **CoMBo Interview Guide**

**PATIENT FACING**

**Objective:** Participants in combined contingency management to reduce stimulant use and optimize ART adherence hold specific insight into their individual needs and program experience. Interviews should seek to elicit participants’ thoughts on:

1. What program components are essential vs. adaptable
2. Perceived efficacy of the program in promoting participation and behavioral change
3. Improvements to promote engagement and overall experience in the program

**CFIR Constructs:**

1. Intervention Characteristics
   - Adaptability
   - Relative Advantage
2. Characteristics of Individual
   - State of change
   - Needs & Priorities
3. Process
   - Engaging
   - Executing
   - Reflecting & Evaluating

**Potential Interviewees:**

- Target n = 15 participants who returned after enrollment
  - Target 50% who attended majority of visits
- Target n = 5 particpants who did not return after enrollment

**Proposed Questions:**

*Plan for ~1 hour-long interviews*

KEY

- ** CFIR Constructs:*
  - *InT = intervention characteristics*
  - *InD = characteristics of the individual*
  - *P = process*

| *#* | *Question* | *CFIR Construct(s) ** | *Obj* |
| --- | --- | --- | --- |
| 1 | How would you describe the CoMBo program to a friend? |  |  |
| 2 | Why did you participate in the program? | InD: state of change | 2 |
| 3 | What was your overall sense of how the program affected your life, if at all? | P: executing | 2 |
| 4 | What things did you like about the program?  *Prompt: examples include relationships with program staff, gift cards for ART and stimulants, once weekly visits, one-one-one, etc.* | InT: adaptability | 1 |
| 5 | What things did you not like about the program?  *Prompt: some examples could be relationships with program staff, gift cards for ART and stimulants, once weekly visits, one-one-one, etc.* | InT: adaptability | 1,3 |
| 6 | What was your experience earning gift cards for both negative stimulant tests and positive HIV medication (ART) tests?  *Prompt if participant struggling to answer: What were your thoughts about the gift cards offered? Would you change anything about the gift cards?* | P: executing | 1 |
| 7 | How would your experience be different if we didn’t give gift cards for taking HIV medication? | P: executing | 1 |
| 8 | How would your experience be different if you had to come in 2 or 3 times per week? | InT: relative advantage |  |
| 9 | What difficulties did you face in your life during the program?  *Follow up: How did this difficulties impact your engagement in the program?* | P: engaging | 1,3 |
| 10 | What changes would you make to help people participate in the program? | P: engaging | 3 |
| 11 | What do you think about the program being offered in the clinic?  *Follow up:* Would you have liked it better if offered somewhere else like an addiction clinic or a harm reduction organization? | InT: relative advantage | 1 |
| 12 | Did the program help you create changes in your life?  *Follow up:* Would you be open to providing some specific examples? | P: reflecting & evaluating | 2 |
| 13 | Did these changes continue after you graduated?  *Follow up:* Why or why not? | InD: needs and priorities | 3 |
| 14 | What are ways we could help make these changes last beyond the program for you? What, if any, behavior changes lasted beyond your time in the program? | P: reflecting & evaluating | 3 |
| 15 | What downsides were there to participating? | P: reflecting & evaluating | 2 |
| 16 | Would it help you to participate in a program like this again in the future?  *Follow up:* How would it help you? | P: reflecting & evaluating | 3 |

##### 
