# Supplementary material for: Harm Reduction Contingency Management for Stimulant Use Reduction and Antiretroviral Therapy Adherence in HIV Primary Care: Protocol for an Implementation Effectiveness Study
Source: JMIR Res Protoc. 2025 Aug 18;14:e67292. doi: 10.2196/67292 (PMC12402737; doi:10.2196/67292)
Supplement: Multimedia Appendix 3 [file resprot_v14i1e67292_app3.docx]

**STAFF FACING**

**Objective:** Staff holds valuable insight into the culture and infrastructure of the intervention setting (W86), as well as insight into population-level needs and subsequent clinic priorities. Interviews should seek to elicit staff perception of:

1. the intervention’s compatibility with the setting’s values and existing processes
2. how well the intervention meets patient needs, and the relative priority of these needs
3. changes to improve the intervention and/or promote its long-term sustainability

**CFIR Constructs:**

- Inner Setting
  - Networks & Communication
  - Tension for Change
  - Compatibility
  - Relative Priority
- Characteristics of the Individual
  - Knowledge & Beliefs about the Intervention

**Potential Interview-ees:**

- Heneliaka (Nurse, Women’s clinic)
- Alberto (SW, Women’s clinic)
- Liz (MD, POP-UP)
- Christy and/or Caycee (Nurse, POP-UP)
- ~~Hiroki or Joi or Rodrigo (SW, POP-UP)~~
- Front desk? (both clinics)
- Erica Perez (Women’s Clinic)
- Valerie Gruber (Women’s Clinic)
- Jenine Rojas (Women’s clinic)
- ~~Gaby (intervention coordinator)~~

**Proposed Questions:**

- *Plan for 1 hour-long interviews => Max of 15 questions*

KEY

- ** CFIR Constructs:*
  - *IS = inner setting*
  - *KB = knowledge & beliefs [subset of Indvidual Characteristics]*

| *#* | *Interview Question* | *CFIR Construct(s) ** | *Obj* |
| --- | --- | --- | --- |
| 1 | How compatible was the intervention with existing work processes at the clinic? What synergies and challenges did you observe? | *IS: compatibility* | *1* |
| 2 | What suggestions do you have for improving the intervention’s compatibility with existing work processes? | *IS: compatibility* | *1* |
| 3 | How have you referred patients? (Thoughts on email referrals vs on-site?) | *IS: networks* | *1* |
| 4 | What changes you would recommend to enhance participant engagement? | *KB* | *1* |
| 5 | How do you feel about the intervention being used at [Women’s clinic or POP-UP]? | *KB* | *1* |
| 6 | What clinic and/or patient needs were met by the intervention? | *KB* | *2* |
| 7 | What changes did participants experience a result of their involvement? | *KB* | *2* |
| 8 | In your perception, who are the types of patients that benefited from the intervention? | *KB* | *2* |
| 9 | Is there a need for this intervention? | *IS: tension for change* | *2* |
| 10 | How else would these needs be met without the intervention? | *IS: tension for change* | *2* |
| 11 | How important is it to implement this intervention compared to the other priorities? | *IS: relative priority* | *3* |
| 12 | Would you like to see the intervention stay long-term? | *KB* | *3* |
| 13 | What changes need to happen for you to support the implementation of this program long-term? | *KB* | *3* |
