# Supplementary material for: Harm Reduction Contingency Management for Stimulant Use Reduction and Antiretroviral Therapy Adherence in HIV Primary Care: Protocol for an Implementation Effectiveness Study
Source: JMIR Res Protoc. 2025 Aug 18;14:e67292. doi: 10.2196/67292 (PMC12402737; doi:10.2196/67292)
Supplement: Multimedia Appendix 4 [file resprot_v14i1e67292_app4.doc]

**UNIVERSITY OF CALIFORNIA, SAN FRANCISCO**

**CONSENT TO PARTICIPATE IN A RESEARCH STUDY**

**Title: CoMBo Study – Contingency Management for Both HIV And Stimulant Use**

| Principal Investigator: | Ayesha Appa, MD  UCSF Assistant Professor of Medicine  995 Potrero Avenue, Building 80  Box 0874, San Francisco, CA 94110  email: ayesha.appa@ucsf.edu |
| --- | --- |

This is a research study about a new program delivering care to people living with HIV who use stimulants, interested in reducing substance use and supporting HIV medication adherence. The study researchers, Drs. Ayesha Appa, Monica Gandhi, Phillip Coffin, Kelly Knight, Elise Riley and/or Gabriel Chamie, from the UCSF Department of Medicine will explain this study to you.

Research studies include only people who choose to take part. Please take your time to make your decision about participating, and discuss your decision with your family or friends if you wish. If you have any questions, you may ask the researchers.

You are being asked to take part in this study because you have expressed interest in cutting down stimulants and receiving HIV medication adherence support at Ward 86 Positive Health Program.

**Why is this study being done?**

The CoMBo study is a new program dedicated to providing support for people who use stimulants and need prolonged infection treatment in the hospital and attached nursing facility (4A). The purpose of this study is to understand whether this program works well for our patients and staff.

**Who pays for this study?**

The CoMBo study is funded by an NIH grant to UCSF called Building Interdisciplinary Careers in Women’s Health (BIRCWH).

**What is the usual care for my condition?**

This study provides additional support for HIV medication adherence, but usual care for your HIV as dictated by your primary care provider will not be affected whether or not you enroll in this study. Usual care for stimulant disorder is varied, but can involve medications, counseling, or residential treatment. Study enrollment will not influence your ability to access any of these things, though if you do go into residential treatment, we will pause study participation.

**How many people will take part in this study?**

Between 20-50 people will take part in this study.

**What will happen if I take part in this research study?**

If you agree to participate, the following procedures will occur:

- You will be invited to come to Ward 86 once per week for contingency management, which is a behavioral program that provides incentives for meeting your treatment goals (i.e., in this case, for medication adherence and/or stimulant use reduction).
  - During the first visit, they will ask you about your goals for addiction and infection treatment, in addition to other questions about your life and medical history. If you agree, they will collect a baseline urine and hair sample, and you will draw from a fishbowl until you earn your first incentive/prize. This may take about 30-60 minutes. For the urine sample, you will be asked to collect a sample in a designated clinic restroom. For the hair sample, research staff will collect a small amount of hair from the back of the scalp using clippers (the amount of hair removed will not be noticeable).
  - During the rest of the visits, research staff will analyze a urine sample that you provide in clinic, and you will be eligible to earn increasing rewards if negative for cocaine/methamphetamine and positive for tenofovir (your HIV medication). These visits will be shorter, at most 30 minutes. The two behaviors you will be reward for are (1) absence of stimulants in urine and (2) presence of tenofovir in urine. For each of these behaviors you demonstrate at a given visit, you are eligible for an incentive prize. The number of prizes you get for a given behavior depends on how many times in-a-row you demonstrate the behaviors; for example, if you had NO STIMULANTS in your urine last week and you received 1 prize, today you can get 2 prizes if there are no stimulants in your urine. Your tests should be ready within minutes.
  - If you miss a visit, you are welcome and encouraged to come to the next visit, which will occur the following week. The number of fishbowl draws you are eligible for each behavior will re-set to one when you return after missing a visit.
  - Within one month of completion, the study team will try to contact you by phone or in-person to discuss enrollment in a second part of the study focused on participant feedback on study procedures.
  - None of the interviews will be recorded, but the provider will fill out an online form with you in the room.

**Study location:** All these procedures will be done at the Ward 86 clinic, 995 Potrero Avenue (Building 80), San Francisco CA 94110.

How long will I be in the study?
Participation in this study involves once weekly visits for 12 weeks (maximum ~12-15 hours), including 3 monthly study visits during this time. If you choose to participate in the second part of the study, this will extend your time in the study by up-to one month, for a total of 16 weeks.

Can I stop being in the study?
Yes. **You can decide to stop at any time.** Just tell the study researcher or staff person right away if you wish to stop being in the study.

What side effects or risks can I expect from being in the study?

- We will try to protect your health information as much as possible, but there is a small chance that your health records could be exposed. To protect your privacy, your urine drug test and tenofovir results will not be posted in your medical chart.
- We are collecting a small amount of hair from the back of the head (equivalent to daily hair loss), and while these are simple procedures, theoretically one could harm themselves since clippers are being used.
- Some of the interview questions may make you uncomfortable, though you can decline to answer any questions and/or exit the program at any time.
- While the urine and tenofavir tests we use are very accurate, it is possible for them to return an incorrect result. If you do not believe the test result to be correct, we will discuss this together, but the number of draws from the fishbowl for prizes you are eligible for in a given visit will be determined solely by the test result.
- It is possible that there are unforeseen risks to participating in the study.

Are there benefits to taking part in the study?

- You may or may not benefit from participating in this study.
- We hope that this program will help achieve your goals of reduced drug use and taking HIV medication regularly.
- We want to ensure we are providing the best, non-judgmental care to all people at ZSFG. Your feedback on this program will help us take better care of patients.

What other choices do I have if I do not take part in this study?
Your other choices may include not getting treatment, getting standard treatment for your condition without being in a study, or taking part in another study. If you decide not to take part in this study, there will be no penalty to you. You will not lose any of your regular benefits, and you can still get your care from our institution the way you usually do.

**How will my specimens and information be used?**
Researchers at UCSF will use your specimens and health behavior information to conduct this study. Once the study is done using your specimens and information, we may use the remaining specimens and information collected for future research studies or share them with other researchers so they can use them for other studies in the future. We will not share your name or any other personal information. We cannot guarantee that this will prevent future researchers from determining who you are. We will not ask you for additional permission to share this de-identified information.

Will information about me be kept private?
We will do our best to make sure that the personal information gathered for this study is kept private. However, we cannot guarantee total privacy. Your personal information may be given out if required by law. If information from this study is published or presented at scientific meetings, your name and other personal information will not be used. Authorized representatives from the following organizations may review your research data for the purpose of monitoring or managing the conduct of this study: Representatives of the University of California, San Francisco, Zuckerberg San Francisco General Hospital, and the NIH.

**Certificate of Confidentiality**

This research is covered by a Certificate of Confidentiality from the National Institutes of

Health. This means that the researchers cannot release or use information, documents, or

samples that may identify you in any action or suit unless you say it is okay. They also cannot

provide them as evidence unless you have agreed. This protection includes federal, state, or

local civil, criminal, administrative, legislative, or other proceedings. An example would be a

court subpoena.

There are some important things that you need to know. The Certificate DOES NOT stop

reporting that federal, state or local laws require. Some examples are laws that require

reporting of child or elder abuse, some communicable diseases, and threats to harm yourself or

others. The Certificate CANNOT BE USED to stop a sponsoring United States federal or state

government agency from checking records or evaluating programs. The Certificate DOES NOT

stop disclosures required by the federal Food and Drug Administration (FDA). The Certificate

also DOES NOT prevent your information from being used for other research if allowed by

federal regulations.

Are there any costs to me for taking part in this study?

No. The sponsor has agreed to pay for all items associated with this research study; you or your insurer will not be billed.

Will I be paid for taking part in this study?
In return for your time and effort, you will be paid $25 for each monthly study visit that involves a longer conversation and hair collection (3 total expected).

In addition, financial rewards are a part of the study intervention, so while you will not be additionally paid for participation only, you may be eligible to earn rewards based on meeting your goals to reduce stimulant use and take HIV medication regularly as part of the study procedures as prescribed above. You can earn up to a maximum of $599 total through participation in the study (including hair validation).

**Will I be reimbursed if I pay expenses related to my participation in this study?**You will not be reimbursed for expenses if you take part in this study.

**What happens if I am injured because I took part in this study?**
It is important that you tell your study doctor, Dr. Ayesha Appa, if you feel that you have been injured because of taking part in this study. You can tell the doctor in-person, by phone 628-206-2400, or email ([ayesha.appa@ucsf.edu](mailto:ayesha.appa@ucsf.edu)).

**Treatment and Compensation for Injury:**  If you are injured as a result of being in this study, the University of California will provide necessary medical treatment.  The costs of the treatment may be billed to you or your insurer just like any other medical costs, or covered by the University of California, depending on a number of factors.  The University does not normally provide any other form of compensation for injury.  For further information about this, you may call the office of the Institutional Review Board at 415- 476-1814.

What are my rights if I take part in this study?
Taking part in this study is your choice. You may choose either to take part or not to take part in the study. If you decide to take part in this study, you may leave the study at any time. No matter what decision you make, there will be no penalty to you in any way. You will not lose any of your regular benefits, and you can still get your care from our institution the way you usually do. Conversely, if you are not able to adhere to study procedures, the study Principle Investigator may decide to terminate your participation in the study.

Who can answer my questions about the study?
If you wish to ask questions about the study or your rights as a research participant to someone other than the researchers or if you wish to voice any problems or concerns you may have about the study, please call the Institutional Review Board at 415-476-1814. You may also contact the Principle Investigator by email ([ayesha.appa@ucsf.edu](mailto:ayesha.appa@ucsf.edu)).

A description of this clinical trial will be available on [http://www.ClinicalTrials.gov](http://www.ClinicalTrials.gov/), as required by U.S. Law. This Web site will not include information that can identify you. At most, the Web site will include a summary of the results. You can search this Web site at any time. The National Clinical Trial (NCT) number for this study is *“not yet assigned.”*

# CONSENT

You have been given a copy of this consent form to keep. You will be asked to sign a separate form authorizing access, use, creation, or disclosure of health information about you.

**PARTICIPATION IN RESEARCH IS VOLUNTARY.** You have the right to decline to be in this study, or to withdraw from it at any point without penalty or loss of benefits to which you are otherwise entitled.

If you wish to participate in this study, you should sign below.

Date Participant's Signature for Consent

Date Person Obtaining Consent
