# Supplementary material for: Harm Reduction Contingency Management for Stimulant Use Reduction and Antiretroviral Therapy Adherence in HIV Primary Care: Protocol for an Implementation Effectiveness Study
Source: JMIR Res Protoc. 2025 Aug 18;14:e67292. doi: 10.2196/67292 (PMC12402737; doi:10.2196/67292)
Supplement: Multimedia Appendix 5 [file resprot_v14i1e67292_app5.docx]

**UNIVERSITY OF CALIFORNIA, SAN FRANCISCO**

**CONSENT TO PARTICIPATE IN A RESEARCH STUDY**

**Universidad de California San Francisco**

**Permiso de Participación en Estudio de investigación**

**Title: CoMBo Study – Contingency Management for Both HIV And Stimulant Use**

| Principal Investigator: | Ayesha Appa, MD  UCSF Assistant Professor of Medicine  995 Potrero Avenue, Building 80  Box 0874, San Francisco, CA 94110  email: ayesha.appa@ucsf.edu |
| --- | --- |

Esta investigación es sobre un programa que ofrece tratamiento para personas con VIH que usan estimulantes, que quieren reducir uso de tales y que desean mejorar su adherencia a los medicamentos de VIH. Los investigadores, Drs. Ayesha Appa, Monica Gandhi, Phillip Coffin, Kelly Knight, Elise Riley o Gabriel Chamie del Departamento de Medicina de UCSF, le van a explicar esta investigación.

Investigaciones solo incluyen personas que consienten en participar. Por favor, tome tiempo para considerar su participación. Si tiene preguntas, puede comunicarse con los investigadores.

Se le pide su participación en esta investigación porque ha expresado su deseo de reducir uso de estimulantes y de mejorar su adherencia a medicamentos de VIH.

**¿Por qué hacemos esta investigación?**

La investigación, que se llama el CoMBo Study, es un programa nuevo para apoyar gentes que usan estimulantes y que toman medicamentos para el VIH. El objetivo de esta investigación es entender si este programa funciona bien para nuestros pacientes y equipo profesional.

**¿Quién pagan por esta investigación?**

El CoMBo Study está financiada por el NIH a UCSF, por medio de una iniciativa que se llama Construyendo Careras Interdisciplinario en La Salud de Mujeres (BIRCWH).

**¿Cuál es el tratamiento típico para mi condición?**

Este estudio provee apoyos para adherirse a los tratamientos de VIH, dichos tratamientos que son dirigidos por su doctor no serán afectados por su participación en el estudio.

El tratamiento para el uso de estimulantes es variado, pero implicaría medicamentos, asesoramiento, o tratamiento residencial. participación en esta investigación no limitaría su acceso a esos tratamientos, pero si decide obtener tratamiento residencial, pararemos su participación en esta investigación.

**¿Cuántas personas participarán en la investigación?**

20-50 personal van a participar en esta investigación.

**¿Qué sucede si yo participo en esta investigación?**

Si decide participar, el siguiente procedimiento ocurrirán:

- Sera invitado a venir a Ward 86 una vez cada semana para el Manejo de la Contingencia, que es un programa conductal que brinda recompensas a los clientes por cambios de comportamiento positivos durante el tratamiento (en este caso, por adherencia al medicamentos y/o reducción de estimulantes).
  - Durante la primera visita, preguntáramos sobre sus objetivos de addicion y tratamiente de VIH, además haremos preguntas sobre su historia clínica. Si usted da su consentimiento, recogeremos unas muestras de orina y de pelo, y elegirá un premio. Eso tomará talves 30-60 minutos. Para la muestra de orina, usted ocupara un baño designado en la clinica. Para la muestra de pelo, un miembro de nuestro equipo va a recoger una cantidad pequeña del pelo de la parte posterior de la cabeza; la cantidad de pelo será imperceptible.
  - Durante las visitas restantes, nuestro equipo le pedirá una nueva muestra de orina, y podrá ganar recompensas si su orina no tiene estimulantes o si tiene medicamentos de VIH (especificamente, un parte del medicamento que se llama Tenofavir). El número de recompensas que ganará depende de cuantas veces consecutivas usted demostrará esos comportamientos. Por ejemplo, si no tuvo estimulantes en su orina la semana pasada, hoy podrá ganar dos en vez de uno premios si no hay estimulante en su orina.

- - Si no falta a una visita, será animado a regresar la próxima semana. El número de premios que podrá ganar volverá a uno.
  - Dentro del mes en que terminará con la investigación, nuestro equipo se comunicará por teléfono o en persona para discutir continuación en el segundo parte de esta investigación, que está enfocado en el su opinión sobre el programa.
  - Las entrevistas no serán grabadas, pero el investigador escribirá unas notas durante la entrevista.

**Lugar de Investigación:** Todos los procimentos ocurrirán en la clinica de Ward 86, 995 Potrero Avenue (Building 80), San Francisco CA 94110.

**¿Por cuánto tiempo yo será parte de esta investigación?**

Su participación requerirá visitas cada semana por 12 semanas, incluyendo 3 visitas cada mes durante ese tiempo. Si participará en la segunda parte de la investigación, eso extenderá su participación hasta 16 semanas.

**¿Puedo parar mi participación en esta investigación?**

Si. Podrá parar en cualquier momento. Solo tiene que indicar su deseo de terminar al investigador.

**¿Qué riesgos o efectos secundarios puede tener este estudio?**

Protegeremos su historia medica lo más posible, sin embargo, existe una posibilidad que sus historia sea expuesta. Para proteger su privacidad, su examen de orina y de tenofavir no serán parte de su historial.

Porque estaremos recogiendo una cantidad pequeña de cabello de su cabeza, existe la posibilidad de danos físicos causados por tijeras. La muestra de cabello será equivalente a una pérdida de cabello natural rutinaria.

Algunas preguntas que se le harán pueden incomodarlo. Ud. Puede reusar a contestar estas preguntas y puede salir del programa cuando quiera.

Los exámenes de orina y de Tenofavir son precisos, pero errores pueden ocurrir, Si Ud. Cree que un resultado es incorrecto, se podrá discutir, pero los premios y recompensas son completamente determinados por esto resultados.

¿Cuáles son los beneficios de participar en el programa?

Ud. puede o no, beneficiarse de su participación.

Esperamos que su participación le ayude a reducir su uso de drogas y que lo inspire a tomar sus medicinas de VIH regularmente.

Queremos proveer servicios de una manera profesional y sin juzgado para todos los pacientes de ZSFG. Su evaluación del programa nos ayudara mucho.

**¿Qué opciones tengo si no participo en el programa?**

Ud. Tiene otras opciones que incluyen no recibir tratamiento, recibir tratamiento regular sin tomar parte en el estudio. No hay consecuencias ni retribución si no participa. Seguirá recibiendo servicios medicos.

**¿Como usaran mis muestras?**

Los científicos de UCSF usuran sus muestras y exámenes en el estudio. Una vez el programa acabe, es posible que se usen estas muestras en futuras investigaciones, y que se compartan con otros estudios. Su nombre e información personal no se compartirá. No podemos garantizar que en el futuro nadie puede determinar su identidad. No le pediremos permiso para compartir no-idenficada información.

**¿Sera mi información mantenida en privado?**

Haremos lo posible para mantener privacidad de su información. No Podemos garantizar esto. Si la ley requiere esta información, se le dará. Los resultados del estudio serán presentados en reuniones y conferencias. Representantes de estas organizaciones tendrán acceso a su información con el propósito de administrar o monitorear el estudio: University of California, San Francisco, Zuckerberg San Francisco General Hospital, y el NIH.

**Certificado de Privacidad**

Esta investigación está cubierta por un Certificado de Privacidad de Instituto Nacional de La Salud. Esto significa que no podemos usar su información, documentos, muestras que lo pueden identificar al menos que sea con su consentimiento. Ud. También tiene que consentir si estos se ocupasen de evidencia. Estas protecciones son federales, estatales, locales, civil, criminal, administrativas, legislativas y de otros procedimientos. Un ejemplo es una subpoena.

Hay cosas importantes que necesita saber. El certificado no puede evitar reportes requeridos por leyes federales, estatales y locales. Por ejemplo, reporte de abuse de niños y adultos mayores, enfermedades infecciosa y peligros a su persona.

**¿Hay costo de participación?**

No, todos los gastos están cubiertos por patrocinadores. No se le harán cargos a su seguranza.

**¿Me pagaran por estar en el estudio?**

Se le pagara $25 por cada visita mensual que incluye conversación y colección de muestras.

También tendrá derecho a premios y regalos por completar sus metas de reducir el uso de estimulantes y de tomar medicina para VIH.

**¿Me pagaran por gastos que yo tenga al participar?**

No se le pagar por estos gastos

**¿Que pasa si me lastimo al participar en el estudio?**

Si esto pasara, comuníquese con Dr. Ayesha Appa. Si cree que esto ha pasado hable en persona o por teléfono 628-206-2400, o email ([ayesha.appa@ucsf.edu](mailto:ayesha.appa@ucsf.edu)).

**Tratamiento y Compensación por lesión**

Si resulta lesionado en la investigación, la Universidad de California le dará tratamiento médico. El costo del tratamiento se o mandaran a ud o a su seguranza. La Universidad normalmente no provee compensación por lesiones. Puede llamar a la Institutional Review Board al 415- 476-1814 como otro recurso.

**¿Cuáles so mis derechos al participar en el estudio?**

Su participación es voluntaria. Si decide dejar el estudio, puede hacerlo. No habrá ninguna consecuencia si Ud. Decide parar su participación.

No perderá beneficios y tendrá acceso a servicios de salud sin ningún cambio. Al mismo tiempo si Ud. no puede seguir las reglas del estudio, es posible que sea expulsado del programa.

**¿Quién puede dar información del estudio?**

Si quiere hacer preguntas sobre el estudio, compartir ansiedades con alguien no relacionado al programa o estudio, puede comunicarse con Institutional Review Board al 415-476-1814 o con Principle Investigator por email ([ayesha.appa@ucsf.edu](mailto:ayesha.appa@ucsf.edu)).

*Una descripción del estudio estará disponible en* <http://www.ClinicalTrials.gov> como es requerido por ley. Este sitio no tiene información que lo pueda identificar. La página de web tiene sumario de los resultados. El número del estudio no se la designado y aparece como “*not yet assigned*”

**PERMISO**

Se le ha dado una copia de esta forma para su uso. Se le pedirá que firme la forma para autorizar acceso, uso, y liberar información.

Participación en la investigación es voluntaria. Ud. tiene derecho de rechazar su participación en el estudio y de no seguir en la investigación sin temor a penalidades o a perder servicios.

Si desea participar por favor firme aquí.

Date Participant's Signature for Consent

Date Person Obtaining Consent
